# Supplementary material for: pH Dependence of T2 for Hyperpolarizable 13C-Labelled Small Molecules Enables Spatially Resolved pH Measurement by Magnetic Resonance Imaging
Source: Pharmaceuticals (Basel). 2021 Apr 2;14(4):327. doi: 10.3390/ph14040327 (PMC8067065; doi:10.3390/ph14040327)
Supplement: Supplementary file 1 [file pharmaceuticals-14-00327-s001.pdf]

pH dependence of T_2 for ^{13}C-labelled small molecules enables spatially resolved pH measurement by hyperpolarized magnetic resonance imaging

Martin Grashei [1], Christian Hundshammer [1], Frits H.A. van Heijster [1], Geoffrey J. Topping [1], Franz Schilling [1]*

Affiliations

[1] Technical University of Munich, School of Medicine, Department of Nuclear Medicine, 81675 Munich, Germany

* Corresponding author e-mail: schilling@tum.de

Supplementary Material

S1: Concentration dependence of T_2 for [1-^{13}C]pyruvate and [1-^{13}C]acetate

pH titration of stock solutions involves addition of small volumes of acids and bases, which increases the overall volume of the stock solution and therefore reduces the concentration of the dissolved ^{13}C-labelled compound. In addition, during injection of these ^{13}C-labelled compounds after hyperpolarization, heterogeneous distribution of the bolus leads to local concentration differences in tissue. Therefore, the concentration dependencies of T_2 were assessed (Figure S1).

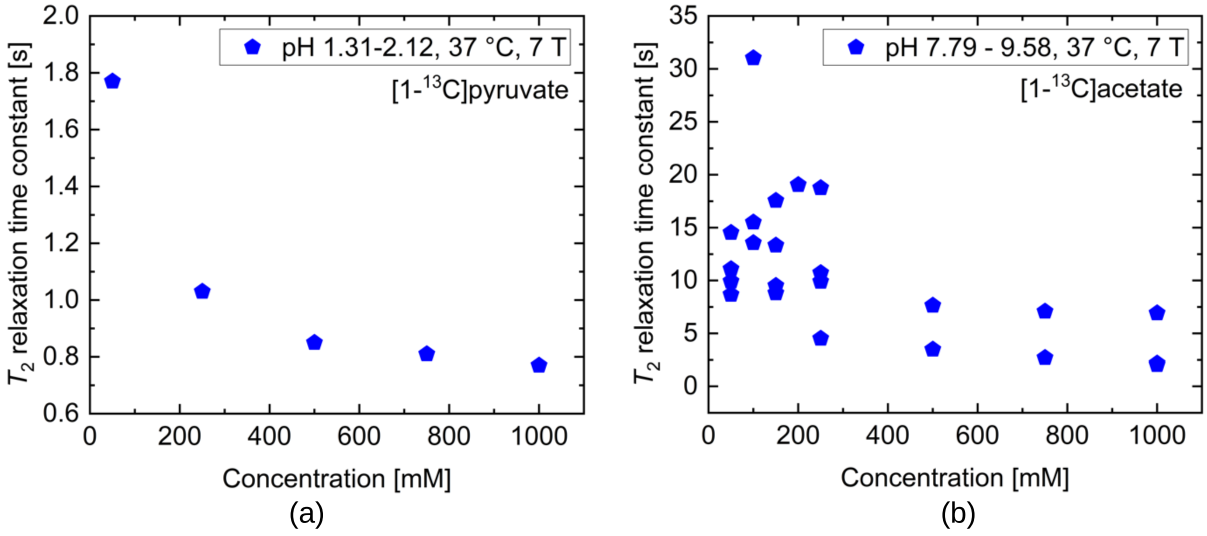

Supplementary Figure S1. T_2 dependence on concentration for [1-^{13}C]pyruvate (a) and [1-^{13}C]acetate (b).

[1-^{13}C]pyruvate shows a monotonic increase of T_2 from higher towards lower concentrations, with a two-fold increase in T_2 from 250 mM to 50 mM. Acetate shows scattering of T_2 at lower concentrations, with a two- to six-fold increase in T_2 values at concentrations below 250 mM. For both molecules, proton exchange might be slowed down at lower concentrations [1], which can most likely be explained by the lower ability to form dimers at higher dilution, therefore rendering hydrogen bonds with the carboxyl groups less effective in contributing to T_2 relaxation. Also, for [1-^{13}C]acetate molecules, it was observed that concentrations below 250 mM result in the distance between hydrated acetate ions becoming too large to still allow hydrogen-bond mediated interaction [2]. This increased distance between acetate-water clusters potentially reduces T_2 relaxation. Consequently, and much like the pH-induced changes in T_2, this strong variation limits the applicability of these compounds for pH imaging *in vivo* by T_2 mapping of their hyperpolarized states.

S2: Magnetic field strength dependence of T_2 for [1-^{13}C]pyruvate

The magnetic field strength dependence of T_2 of [1-^{13}C]pyruvate at different pH values was determined from thermal equilibrium measurements at 7 T and 14.1 T and from hyperpolarized measurements at 1 T after addition of 80 mM TRIS buffer. T_2 of [1-^{13}C]pyruvate exhibits similar behavior for pH variations, with an up to 57% reduction in the long T_2 regime (pH 6 - 9) for 14.1 T compared to 1 T and 7 T. Further decreases due to higher B_0 are also present in the moderately acidic pH (pH 2 – 4) range. Hence, this field-dependent chemical shift anisotropy relaxation mechanism seems to contribute more strongly to T_2 relaxation in pH milieus with reduced conformational change of the molecule or proton exchange of the carboxyl group.

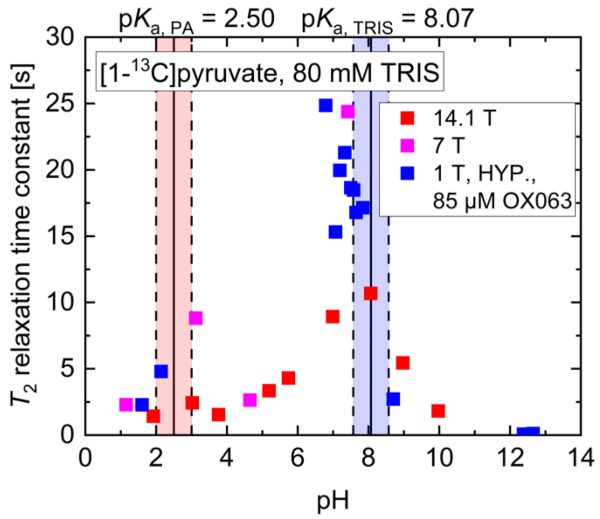

Supplementary Figure S2. T_2 dependence on pH shown for three different magnetic field strengths for [1-^{13}C]pyruvate in H_2O containing 80 mM TRIS and 85 µM OX063 radical for hyperpolarized compounds at 1 T. T_2 of [1-^{13}C]pyruvate shows the strongest reduction with increasing magnetic field at moderately acidic (pH 2 – 4) and slightly alkaline (pH 7 – 9) pH.

S3: Voxel-wise fitting of echo signal decay curves for T_2-mapping of [1-^{13}C]acetate using RARE

For generation of T_2-maps, intensities of echo images were plotted versus effective echo time (160 ms per echo image) and fitted with a mono-exponential function with offset (Figure S3) by minimizing the sum-of-squared residuals between the data points and the fit curve. Fitting was performed in voxels where the initial echoes exceeded a signal-to-noise peak ratio of 20. All fits showed qualitatively good agreement of the data with the applied model.

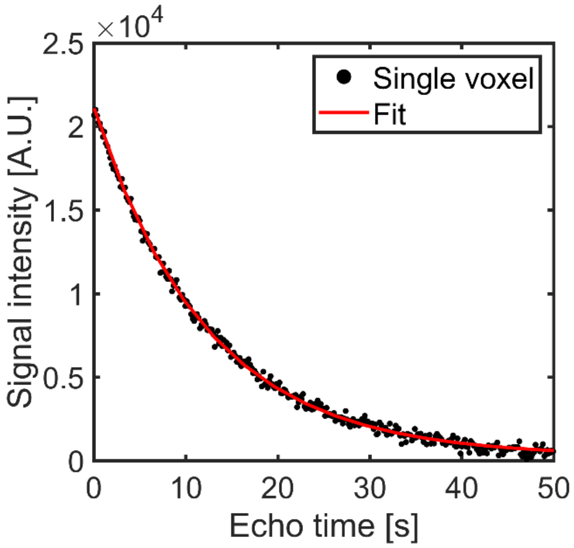

Supplementary Figure S3. Fitting of a single voxel echo decay extracted from echo images curve versus the effective echo time. For representation purposes, signal from echoes at later time points (echo time > 50 s) are not shown in the plot but were included in the fitting process. Acquired data sets showed good agreement with fit curves for all voxels included in the T_2-map shown in Figure 4 c.

S4: Titration protocols

For titrations at 7 T, exemplary titration protocols are listed in the following tables. For each titration step, the resulting pH, the resulting concentration, the resulting ion concentration (either Na^+ or Cl^-) as well as the added volume of acid or base relative to the previous titration step is listed:

Table 1. Titration protocol towards acidic pH values for [1-^{13}C]acetate:

**[1-^{13}C]acetate**	**pH**	**concentration [mM]**	**ion concentration [mM]**	**volume added [µL]**
Titration step 1	4.92	247.65	91.58	95
Titration step 2	4.45	245.94	159.06	70
Titration step 3	3.94	244.62	212.08	55

Titration step 4	3.12	243.90	241	30
Titration step 5	2.45	243.43	260.28	20
Titration step 6	2.00	243.31	265.1	5
Titration step 7	1.48	242.84	284.38	20
Titration step 8	1.05	241.55	337.4	55

Table 2. Titration protocol towards basic pH values for [1-^{13}C]acetate:

[1-^{13}C]acetate	pH	concentration [mM]	ion concentration [mM]	volume added [µL]
Start	9.53	250	0	0
Titration step 1	10.18	249.98	1	1
Titration step 2	10.78	249.95	2	1
Titration step 3	11.44	249.88	5	3
Titration step 4	11.84	249.33	27	22
Titration step 5	12.40	247.60	97	70
Titration step 6	12.68	244.69	217	120

Table 3. Titration protocol towards basic pH values for [1-^{13}C]pyruvate:

[1-^{13}C]pyruvate	pH	concentration [mM]	ion concentration [mM]	volume added [µL]
Start	1.41	250	0	0
Titration step 1	1.14	249	38.56	40
Titration step 2	1.99	246.67	133.56	95
Titration step 3	2.47	245.10	198.56	65
Titration step 4	2.94	243.78	253.56	55
Titration step 5	3.49	243.12	281.56	28
Titration step 6	3.98	242.86	292.56	11
Titration step 7	4.50	242.67	296.76	8
Titration step 8	4.93	242.18	298.76	21
Titration step 9	5.50	241.83	299.51	15
Titration step 10	5.87	241.66	299.83	7
Titration step 11	6.19	240.82	300.23	36
Titration step 12	6.53	240.22	300.53	26
Titration step 13	6.58	240.20	300.58	1
Titration step 14	6.76	240.18	300.62	1
Titration step 15	7.12	240.15	300.68	1
Titration step 16	6.81	240.15	300.68	0
Titration step 17	7.10	240.13	300.73	1
Titration step 18	7.43	240.11	300.78	1
Titration step 19	7.90	240.08	300.83	1
Titration step 20	8.12	240.06	300.88	1
Titration step 21	8.69	240.04	300.93	1
Titration step 22	8.83	240.02	300.98	1
Titration step 23	9.71	239.95	301.13	3
Titration step 24	10.16	239.88	301.28	3
Titration step 25	10.52	239.76	301.53	5
Titration step 26	10.93	239.35	302.23	18
Titration step 27	11.42	239.28	303.50	3

Titration step 28	11.89	239.26	304.50	1
Titration step 29	12.31	239.23	305.50	1
Titration step 30	12.77	239.07	312.50	7
Titration step 31	13.10	238.85	322.50	10
Titration step 32	9.47	238.12	344.28	32

From this table, it can be seen that all titrations altered sample concentration by less than 5% (largest deviation: 11.88 mM reduction for [1-^{13}C]pyruvate, titration step 32). In addition, Na^+ or Cl^--ion concentration was kept below 350 mM for all titration curves, which is still in a range where salt concentration effects on T_2 are of minor importance (see Figure 3 b and d). As an additional control for [1-^{13}C]pyruvate, titration step 32 aimed to titrate the stock solution back to a pH regime close to pyruvate global T_2 maximum. Here, T_2 was assumed to be most sensitive to influences from dissolved ions or alterations in concentration. However, the corresponding data point in Figure 2 b (pH 9.47, T_2 18.72 s) does not show a mismatch compared to the expected T_2 behaviour in this pH range.

S5: Error Estimation for T_2 and pH measurements

To assess the uncertainty of the measured T_2 and pH values, an experimental series as follows was designed: Three samples of 250 mM [1-^{13}C]pyruvate in water (total volume 2 ml) and three samples of 250 mM [1-^{13}C]acetate in water (total volume 2 ml) were prepared independently. Of these, two samples of each compound were measured with three repetitions and one sample of each compound was measured with ten repetitions. All samples were measured at 18.8 ± 0.4 °C. The acquisition of multiple repetitions of a single sample allowed the precision of the T_2 measurement or its stability over time to be evaluated, whereas the measurements of several independently-prepared samples allowed the impact of the reproducibility of sample preparation on pH and T_2 measurement to be evaluated. Accordingly, the pH of each sample was measured before and after each acquisition block.

The results from all measurements are listed in the following two tables:

Table 4. Measured T_2 values of 250 mM [1-^{13}C]pyruvate of three independently prepared samples on which 3 or 10 measurement repetitions were performed.

**[1-^{13}C]pyruvate**	**T_2 [s] sample #1**	**T_2 [s] sample #2**	**T_2 [s] sample #3**	**pH**	**sample #1**	**sample #2**	**sample #3**
repetition 1	2.09	2.06	2.64	before	1.49	1.50	1.49
repetition 2	2.12	2.05	2.66	after	1.52	1.52	1.57
repetition 3	2.09	2.04	2.67	**mean**	**1.51**	**1.51**	**1.53**
repetition 4			2.69				
repetition 5			2.69				
repetition 6			2.64				
repetition 7			2.66				
repetition 8			2.63				

repetition 9			2.60				
repetition 10			2.65				
**mean ± std**	**2.10 ± 0.02**	**2.05 ± 0.01**	**2.65 ± 0.03**				
**#1 - #3 mean ± std**		**2.27 ± 0.33**		**#1 - #3 mean ± std**		**1.52 ± 0.01**	

Table 5. Measured T_2 values of 250 mM [1-^{13}C]acetate of three independently prepared samples on which 3 or 10 measurement repetitions were performed.

**[1-^{13}C]acetate**	**T_2 [s] sample #1**	**T_2 [s] sample #2**	**T_2 [s] sample #3**	**pH**	**sample #1**	**sample #2**	**sample #3**
repetition 1	9.14	10.53	13.75	before	9.22	9.34	9.07
repetition 2	8.76	9.85	13.33	**mean ± std** (before)		9.21 ± 0.14	
repetition 3	8.42	9.18	12.97				
repetition 4			12.64	after	7.11	7.67	7.64
repetition 5			12.21	**mean ± std** (after)		7.47 ± 0.32	
repetition 6			12.03				
repetition 7			11.78				
repetition 8			11.53				
repetition 9			11.29				
repetition 10			11.13				
**Drift rate [s/min]**	**-0.07**	**-0.14**	**-0.06**				
**#1 - #3 mean drift rate [s/min]**		**-0.09 ± 0.04**		**#1 - #3 mean ± std**		**8.34 ± 0.98**	

For [1-^{13}C]pyruvate, repetitions of T_2 measurements on the same sample show very good agreement between T_2 values (Table 1, left side) with absolute variations of less than 0.1 s and standard deviations between 0.01 to 0.03 s. Also, the measured T_2 values did not show any drifts as there was no systematic change of T_2 as a function of repetitions (Figure S4 a). However, sample #3 showed an elevated T_2 relaxation time constant, which might be attributed to a slightly elevated pH compared to samples #1 and #2. Nevertheless, sample pH stayed almost constant for all samples during the measurement as indicated by the small differences between pH measurements before and after the T_2 measurements (Table 1, right side). In addition, averaging of all mean T_2 values of each sample results in a standard deviation of 0.33 s, which is mainly attributed to the differing T_2 values measured on sample #3 while the pH standard deviation calculated from mean pH values of all three samples being 0.01 pH units shows good reproducibility in sample preparation.

For [1-^{13}C]acetate, considerable pH drifts occur during multiple repetitions of T_2 measurements, as indicated by deviating pH measurements before and after the T_2 measurement (Table 2, right side), which is also supported by a monotonically decreasing measured T_2 as a function of repetitions (Figure S4 b). We observed this trend for all three prepared samples. As repetitions are spaced by exactly five minutes, drift rates of T_2 can be calculated as changes in seconds in T_2 over one minute [s/min]. Here, comparable drift rates between samples can be observed (Table 2, left side) which depending on the number of repetitions (3 – 10) represent 15 to 30 minutes elapsing time. Nevertheless, at least pH values

before T_2 measurements of independently prepared samples show good agreement between each other indicated by a standard deviation of 0.14 pH units. As individual T_2 measurements as reported in the titration curves in the manuscript slightly varied regarding the time schedule, the observed drifts might be the main reason for the observed data scattering in Figure 2 a.

Overall, the results from this experimental series reflect the challenges in T_2 measurements under varying pH conditions explaining the data scattering as observed in Figure 2, especially for acetate in the pH range 7.11 – 9.22.

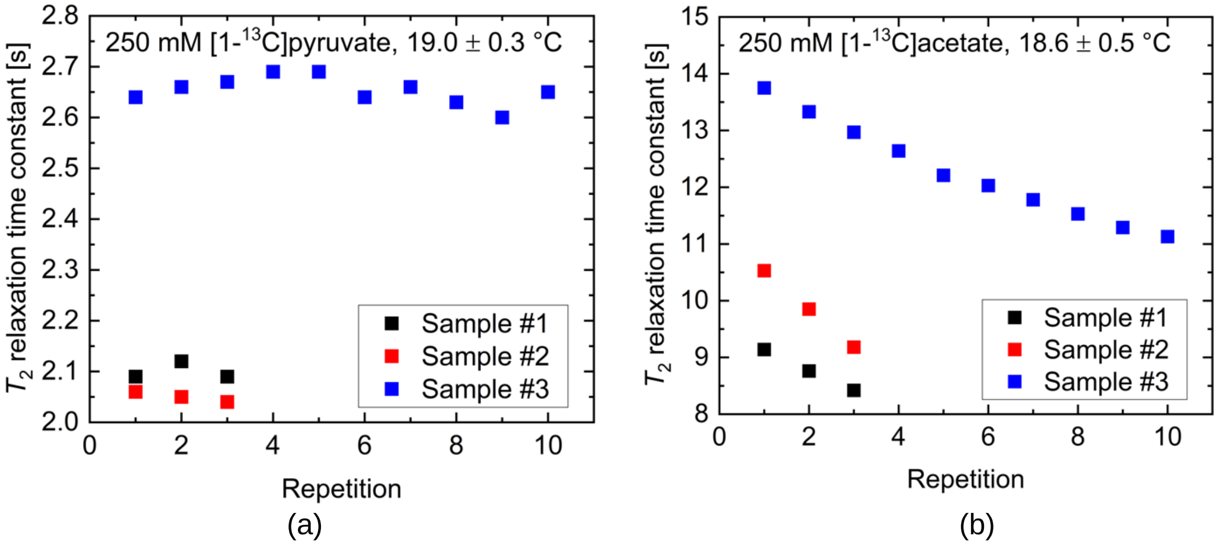

Figure S4: T_2 values measured on three independently prepared samples #1 - #3 of 250 mM [1-^{13}C]pyruvate (**a**) and [1-^{13}C]acetate (**b**) which are reported with the sample mean pH from pH measurements before and after the T_2 measurements. While measurements on [1-^{13}C]pyruvate show high reproducibility, measurements on [1-^{13}C]acetate suffer from drifts in pH- and, consequently, T_2-values.

In summary, these experiments demonstrate that CPMG-acquisitions allow individual measurement of T_2 relaxation time constants with two significant digits after the comma in cases where the sample pH is stable. In these cases, where the pH is sufficiently stable for repeated measurements, such as for pyruvate, the standard deviations from iterative measurements did not exceed 0.05 s. However, larger uncertainties arise from the sample preparation, the resulting pH value and pH changes over time. Here, even for samples with stable pH values, such as for pyruvate, multiple preparations of independent sample cause variations in T_2 leading to an overall standard deviation of 0.33 s. In contrast, for acetate, pH drifts of roughly 0.1 s/min in observed T_2 values due to drifts in pH also limit the precision of the reportable data points. However, a general statement on the errors on T_2 measurement covering all compounds is not possible, as it is determined by the uncertainty in pH which varies between < 0.05 and > 1 pH unit for different compounds. Following the analysis here, we report T_2 values throughout the manuscript with an uncertainty of 0.1 s.

1. Luz, Z.; Meiboom, S. Kinetics of Proton Exchange in Aqueous Solution of Acetate Buffer. *J. Am. Chem. Soc.* **1963**, *85*, 3923-3925, doi:10.1021/ja00907a004.
2. Rahman, H.M.; Hefter, G.; Buchner, R. Hydration of formate and acetate ions by dielectric relaxation spectroscopy. *J Phys Chem B* **2012**, *116*, 314-323, doi:10.1021/jp207504d.
